# 

**Published:** 1880-12

**Authors:** 


### BOOK NOTICES.

In the Standard Series, I. K. Funk & Co., New York, have successfully inaugurated a plan for the publication of the world's best books at about one-tenth their former prices. They have already issued 45 volumes on this plan, and others are weekly added to this list of standard books. The marvelous changes they have wrought in the prices of books is illustrated by the fact that these 45 volumes formerly sold for $60 to $100 ; but, *in the Standard Series, they cost but little over* $9, *or* 10 *to* 30 *cents apiece.*

The poor laboring-man is no longer compelled to take home the "Dime Novel," or let his family do without reading. This publication of good books, in cheap but durable form, opens the way to a great but needed reform. Says Anthony Comstock, "Bad books are working more harm than intemperance—tenfold more ;" and yet we are comparatively indifferent to this stupendous evil.

Our space will permit us to mention but a few of the books which are published in this series. Our readers will do well to send for a complete list. Knight's great "History of England" is complete in eight numbers for $2.80, including postage. Dean Farrar's "Life and Work of St. Paul" is issued in two numbers for 25 cents each. The celebrated "Life of Christ," by the same author, is sold also for half a dollar. A valuable book "On Self Culture," by Prof. Blackie, of Edinburgh, is priced at 10 cents, and Ruskin's "Letters to Workmen" now sells for 30 cents. An interesting life of Rowland Hill, the eccentric English preacher ; Canon Kingsley's "Town Geology ;" Spurgeon's new "John Ploughman's Pictures ;" "The Salon of Madame Necker," a translation of a recent French work, in two parts ; "Mister Horn and His Friends," a book about givers and giving, finely illustrated ; and the celebrated "Thoughts of Marcus Aurelius," translated by George Long ; each costs the reader *but* 15 *cents.* A new book, "Outdoor Life in Europe," by Rev. E. P. Throing ; another, "America Revisited," by George A. Sala, a well-known English writer, are entertaining books for 20 cents each. Leland's translation of Demosthenes' Orations is complete in two parts, and at the same price. Many other books equally instructive may be found in the series. We commend the Standard Series to all book-readers.

"The Beef Bonanza, or How to get rich on the Plains," is the striking title of a somewhat enthusiastic book in relation to raising cattle, sheep and horses in the Great West, by General Brisbin, of the U. S. Army. It embodies a mass of interesting facts and figures accumulated during the author's residence in the West. It contains much matter which will greatly interest the general reader; to the agriculturist contemplating either of the occupations treated of, it will prove a mine of valuable information. Many instances of successes achieved by the managers and owners of western ranches are given. If these represent a fair average of the profits of western stock raising, the wonder is that the business is not speedily over-done. A somewhat careful perusal of the book has left the impression that the gallant author has made out the best case possible for the stock raiser, and that he has dwelt too little upon the risks and drawbacks attending this as they do every business. Yearly profits of thirty to fifty per cent. are certainly very tempting, but there can be no doubt of the correctness of the figures quoted by the author, and many of his facts have been furnished by two gentlemen of high character, who are personally known to us.

The Great West is certainly fortunate in having secured the advocacy of Gen. Brisbin. We regret that he has favored his readers with but brief notices of stock raising in Texas. If this business is so profitable in the colder regions of the north, where expenses for winter food and shelter figure largely in the expense accounts, how much more profitable it must be in a State where winter feeding and shelter are unnecessary. At this time, when the current of emigration is setting so strongly toward Texas, which is preëminently *the* great stock raising State of the Union, the omission referred to is to be regretted.

No one contemplating the formation of a stock farm anywhere should fail to read Gen. Brisbin's book.

Bricks without Straw.—A novel. By Albion W. Tourgee, author of "A Fool's Errand," etc. 12mo. With Frontispiece Illustration. New York: Fords, Howard & Hulbert. 1880. The literature of our country has just been enriched by a new work from the pen of this most popular writer. The philosophical wit and political wisdom of "A Fool's Errand" are still fresh and influential in the public mind, and every one will be glad to welcome a new book by the same author.

---

"When I die," said a married man, "I want to go where there is no snow to shovel." His wife said she presumed he would.

